# Supplementary material for: Does Green Exfoliation of Graphene Produce More Biocompatible Structures?
Source: Pharmaceutics. 2023 Mar 20;15(3):993. doi: 10.3390/pharmaceutics15030993 (PMC10051938; doi:10.3390/pharmaceutics15030993)
Supplement: Supplementary file 1 [file pharmaceutics-15-00993-s001.zip › pharmaceutics-2244740-supplementary.pdf]

Supplementary Materials

Does Green Exfoliation of Graphene Produce More Biocompatible Structures?

Eirini Papanikolaou [1,2,3,*], Yannis V. Simos [1,2,*], Konstantinos Spyrou [2,4], Michaela Patila [2,5], Christina Alatzoglou [5], Konstantinos Tsamis [1,2], Patra Vezyraki [1], Haralambos Stamatis [2,5], Dimitrios P. Gournis [2,4], Dimitrios Peschos [1,2] and Evangelia Dounousi [2,3]

1 Department of Physiology, Faculty of Medicine, School of Health Sciences, University of Ioannina, 45110 Ioannina, Greece
2 Nanomedicine and Nanobiotechnology Research Group, University of Ioannina, 45110 Ioannina, Greece
3 Department of Nephrology, Faculty of Medicine, School of Health Sciences, University of Ioannina, 45110 Ioannina, Greece
4 Department of Materials Science and Engineering, University of Ioannina, 45110 Ioannina, Greece
5 Biotechnology Laboratory, Department of Biological Applications and Technologies, University of Ioannina, 45110 Ioannina, Greece
* Correspondence: epapanikolaou@uoi.gr (E.P.); isimos@uoi.gr (Y.V.S.); Tel.: +30-2651-007-735 (E.P.); Tel.: +30-2651-007-602 (Y.V.S.)

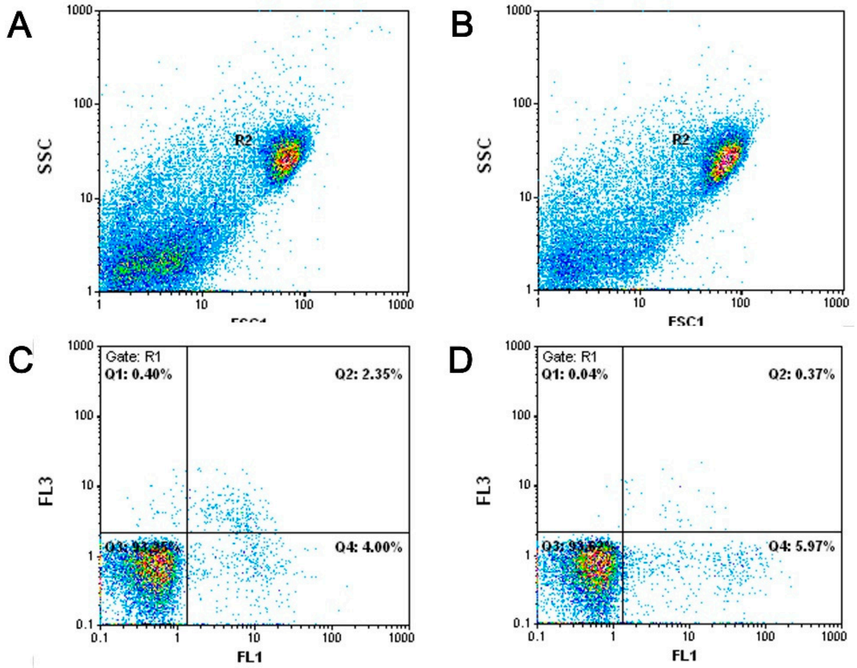

Figure S1. Apoptosis assay using flow cytometry after staining with annexin V-FITC/propidium iodide (PI) in NIH/3T3 cells. Forward versus side scatter (FSC vs SSC) gating was used to identify the cell size and granularity of the untreated (A) and treated with 0.75% v/v DMF for 24h (equal to treatment with 50 μg/mL cG) (B) cells. Representative scatter plots of PI (y-axis) vs. annexin V (x-axis) of the untreated (C) and treated with 0.75% v/v DMF for 24h (equal to treatment with 50 μg/mL cG) (D) cells.

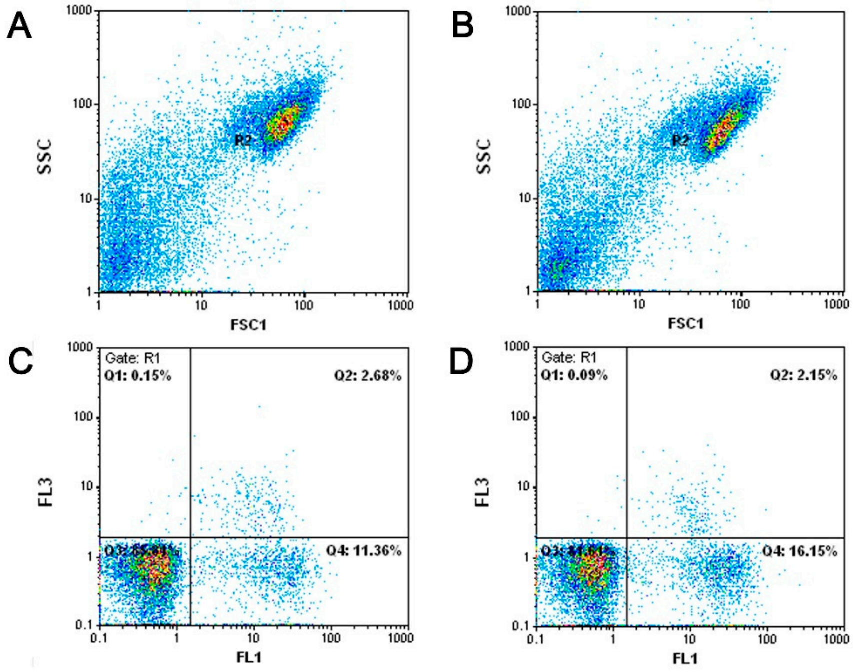

Figure S2. Apoptosis assay using flow cytometry after staining with annexin V-FITC/propidium iodide (PI) in HaCaT cells. Forward versus side scatter (FSC vs SSC) gating was used to identify the cell size and granularity of the untreated (A) and treated with 0.75% v/v DMF for 24h (equal to treatment with 50 μg/mL cG) (B) cells. Representative scatter plots of PI (y-axis) vs. annexin V (x-axis) of the untreated (C) and treated with 0.75% v/v DMF for 24h (equal to treatment with 50 μg/mL cG) (D) cells.

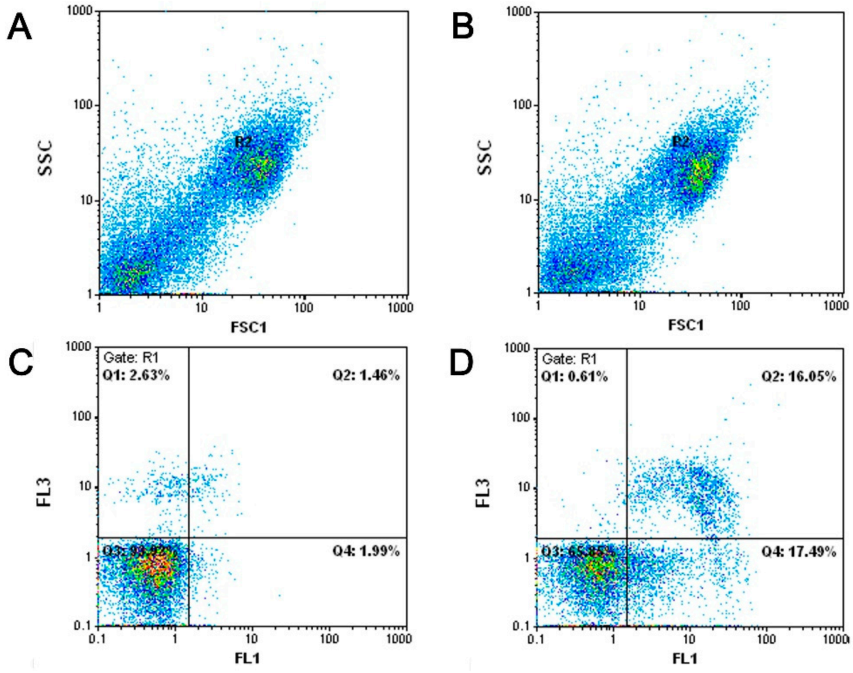

Figure S3. Apoptosis assay using flow cytometry after staining with annexin V-FITC/propidium iodide (PI) in THP-1 derived macrophages. Forward versus side scatter (FSC vs SSC) gating was used to identify the cell size and granularity of the untreated (A) and treated with 0.75% v/v DMF for 24h (equal to treatment with 50 μg/mL cG) (B) cells. Representative scatter plots of PI (y-axis) vs. annexin V (x-axis) of the untreated (C) and treated with 0.75% v/v DMF for 24h (equal to treatment with 50 μg/mL cG) (D) cells.

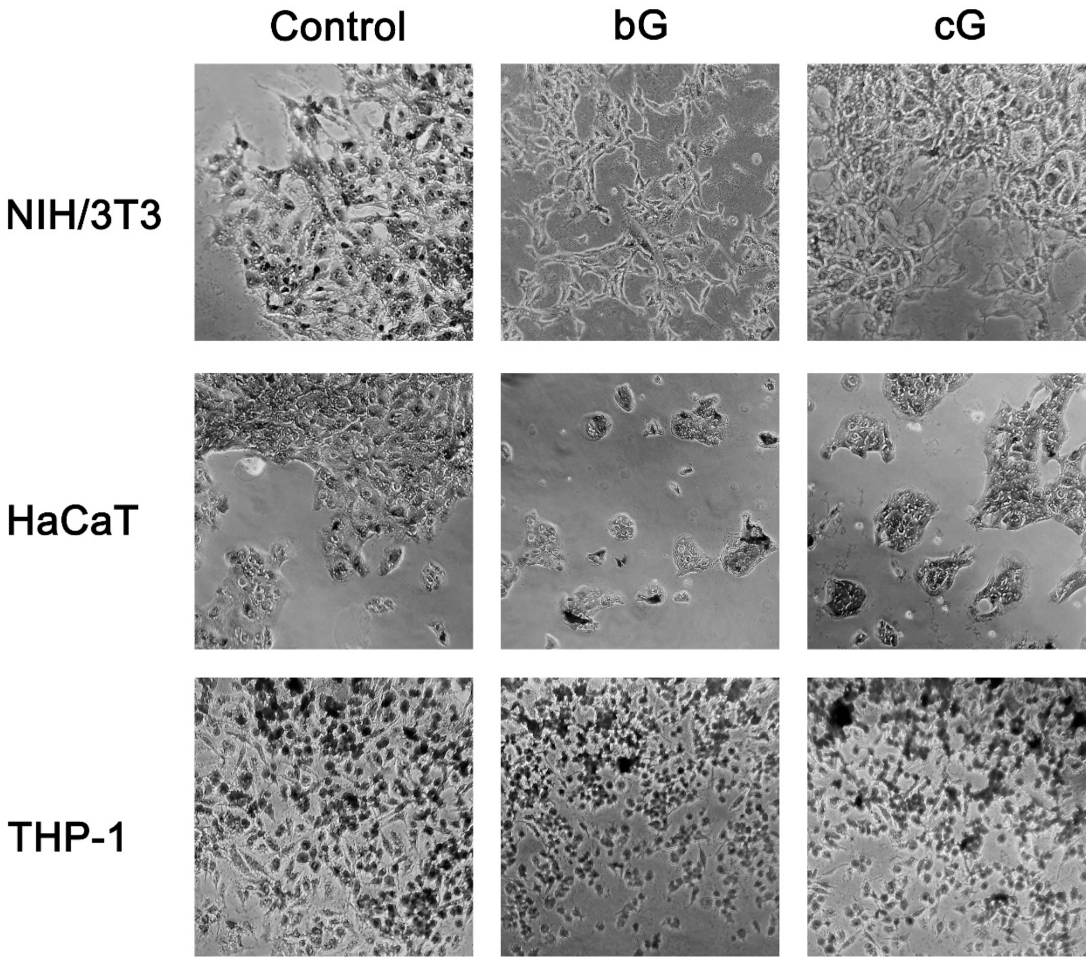

Figure S4. Optical cell images of NIH/3T3, HaCaT cells and THP-1- derived macrophages, after staining with 0.5% crystal violet. Cells were treated with 20 μg/mL of either bG or cG for 24h.

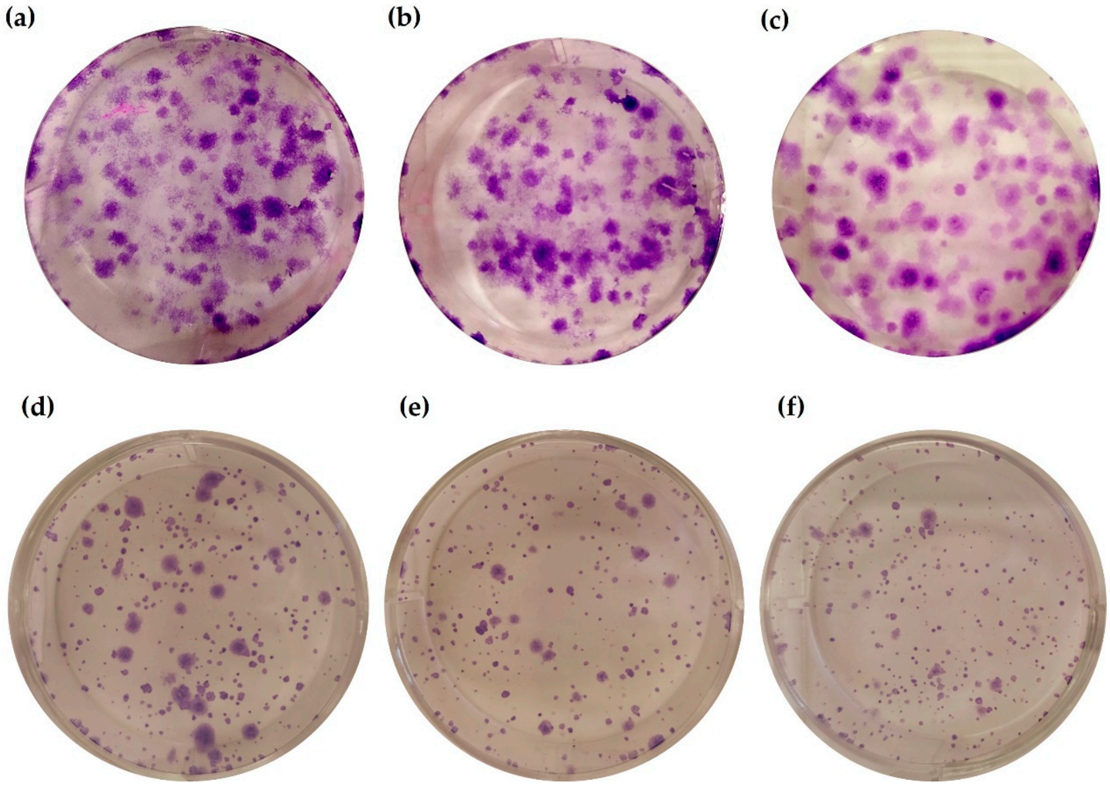

Figure S5. Clonogenic assay in NIH/3T3 (a, b, and c) and HaCaT cells (d, e, and f). Untreated cells (a and d). Cells treated with 20 μg/mL bG (b and e) or cG (c and f) for 48h.
